# The Bacterial Product Violacein Exerts an Immunostimulatory Effect Via TLR8

**DOI:** 10.1038/s41598-019-50038-x

**Published:** 2019-09-20

**Authors:** Francisco A. Venegas, Gabriele Köllisch, Kerstin Mark, Wibke E. Diederich, Andreas Kaufmann, Stefan Bauer, Max Chavarría, Juan J. Araya, Alfonso J. García-Piñeres

**Affiliations:** 1 Escuela de Química, Universidad de Costa Rica, 11501-2060 San José, Costa Rica; 2 Centro de Investigaciones en Productos Naturales (CIPRONA), Universidad de Costa Rica, 11501-2060 San José, Costa Rica; 3 Centro de Investigación en Biología Celular y Molecular (CIBCM), Universidad de Costa Rica, 11501-2060 San José, Costa Rica; 4 Present Address: Institute for Immunology, Philipps-University Marburg, BMFZ, 35043 Marburg, Germany; 5 Department of Parasitology, Philipps University Marburg, 35043 Marburg, Germany; 6 Department of Pharmaceutical Chemistry and Center for Tumor Biology and Immunology (ZTI), Philipps University Marburg, 35043 Marburg, Germany; 7 Core Facility Medicinal Chemistry, Philipps University Marburg, 35043 Marburg, Germany; 8 Institute for Immunology, Philipps-University Marburg, BMFZ, 35043 Marburg, Germany; 9 Centro Nacional de Innovaciones Biotecnológicas (CENIBiot), CeNAT-CONARE, 1174-1200 San José, Costa Rica

**Keywords:** Assay systems, Target identification, Monocytes and macrophages, Transcriptomics

## Abstract

Violacein, an indole-derived, purple*-*colored natural pigment isolated from *Chromobacterium violaceum* has shown multiple biological activities. In this work, we studied the effect of violacein in different immune cell lines, namely THP-1, MonoMac 6, ANA-1, Raw 264.7 cells, as well as in human peripheral blood mononuclear cells (PBMCs). A stimulation of TNF-α production was observed in murine macrophages (ANA-1 and Raw 264.7), and in PBMCs, IL-6 and IL-1β secretion was detected. We obtained evidence of the molecular mechanism of activation by determining the mRNA expression pattern upon treatment with violacein in Raw 264.7 cells. Incubation with violacein caused activation of pathways related with an immune and inflammatory response. Our data utilizing TLR-transfected HEK-293 cells indicate that violacein activates the human TLR8 (hTLR8) receptor signaling pathway and not human TLR7 (hTLR7). Furthermore, we found that the immunostimulatory effect of violacein in PBMCs could be suppressed by the specific hTLR8 antagonist, CU-CPT9a. Finally, we studied the interaction of hTLR8 with violacein *in silico* and obtained evidence that violacein could bind to hTLR8 in a similar fashion to imidazoquinoline compounds. Therefore, our results indicate that violacein may have some potential in contributing to future immune therapy strategies.

## Introduction

**Figure 1:**
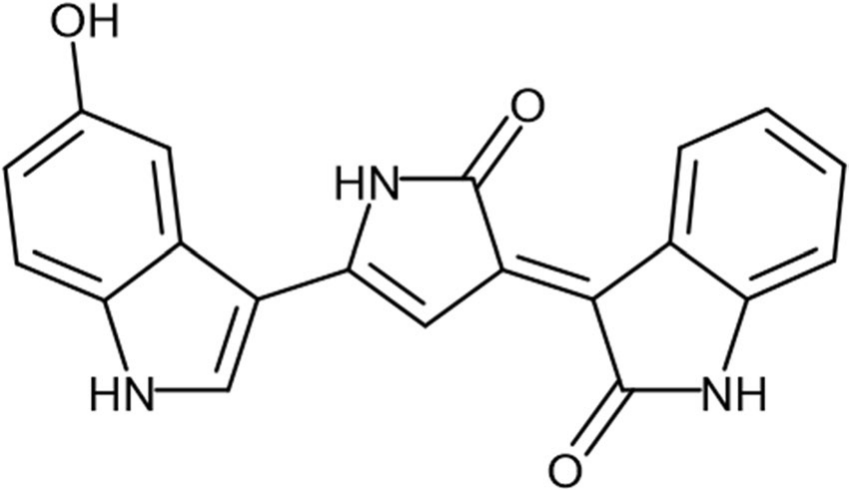
Chemical structure of violacein (3-(1,2-dihydro- 5-(5-hydroxy-1H-indol-3-yl)-2-oxo-3H-pyrrol-3-ilydene)-1,3-dihydro-2H-indol-2-one).

Violacein (Fig. 1) is a natural purple pigment produced by several Gram-negative bacteria^1^, such as *Chromobacterium violaceum*^2^ and *Janthinobacterium lividum*^3^. This secondary metabolite is an alkaloid formed by three structural units: a 5-hydroxyindole, an oxindole and a 2-pyrrolidone^4^. A variety of biological activities have been reported for this compound, including antibacterial^1,5^, anti-viral^6^, anti-inflammatory^7^, antitumor^8^, antileukemic^9^, as well as antifungal, antiparasitic, antiprotozoal, antioxidant and antiulcerogenic^1^. This wide range of biological activities has attracted interest to understand its mechanism of action with the purpose of finding a potential application as a therapeutic agent.

The effect of violacein on immune cells and inflammation has been previously studied. For instance, it was reported that violacein exhibits immunosuppressive, analgesic and antipyretic effects in mice and rats^7^. In another study, violacein showed a gastroprotective effect in rats mediating the maintenance of the balance between pro- and anti-inflammatory cytokines and inhibiting TNF-α production^10^. Violacein also showed anti-inflammatory and anti-tumor activity through inhibition of metalloproteinase in the MCF-7 breast cancer cell line^11^. In a recent work^12^, it was shown that violacein treatment in a mouse model of acute inflammation was able to modulate production of several cytokines: IL-6 and TNF-α levels were reduced, and IL-10 levels were increased compared to untreated mice. A cytotoxic effect towards macrophages was also observed. Moreover, violacein treatment in a mouse model of experimental autoimmune encephalomyelitis (EAE) led to an amelioration of symptoms compared to placebo-treated mice. This study found that violacein exerts this effect by increasing regulatory T-cell counts^12^.

In contrast to the above-mentioned studies suggesting that violacein has an inhibitory effect on TNF-α expression, other studies related to the mechanism of the antileukemic activity of violacein in HL60 cells have found an increase in the pro-inflammatory cytokine TNF-α and the activation of TNF receptor 1 signaling upon incubation with violacein. These results suggest that violacein induces apoptosis of immune cells by TNFR1 activation^9^. Further work by Antonisamy *et al*.^13^ reports that violacein induces apoptosis in human breast cancer cells through upregulation of TNF-α expression and the p53-dependent mitochondrial pathway.

In summary, the evidence shows that violacein exerts an anti-inflammatory effect *in vivo* that involves a reduction in TNF-α production, but these results appear to be in contradiction with some *in vitro* results, where an induction of the pro-inflammatory cytokine TNF-α is observed.

TNF-α is a pro-inflammatory cytokine that is produced by a wide range of cells and regulates several processes like cell proliferation, differentiation and apoptosis^14^. TNF-α signaling is mediated by binding to one of two receptors, TNFR1 or TNFR2^15^. TNFR1 is expressed in most tissues while TNFR2 is exclusively found in immune cells^15^. TNF-α signaling through TNFR1 involves induction of apoptosis, and this implies recruitment of the adaptor protein FADD and caspase-8^16^. TNFR1 also induces nuclear translocation of NF- κB, a transcription factor that promotes cell survival through the induction of anti-apoptotic and inflammatory gene expression^15,16^. TNF-α is relevant to physiological processes such as apoptosis and inflammation, and it has been shown that violacein affects TNF-α expression. However, the evidence of induction or suppression of TNF-α by violacein is not clear. Therefore, further work is needed to clarify the role of this cytokine in the activity of violacein on cells^9,11–13^.

Macrophages are cells of the innate immune system and are able to detect invading microorganisms by recognizing associated molecular patterns (PAMPs) through pattern recognition receptors (PRR) such as Toll-like and NOD-like Receptors (TLR and NLR)^17,18^. Binding of PAMPs to their cognate receptor leads to the production of several mediators of inflammation, such as inflammatory cytokines and chemokines. As a consequence, activation of macrophages and phagocytes leads to chemotaxis of immune cells, inflammation and activation of an adaptive response, with a global effect of protection of the organism against microbes^18^.

In this study, we investigated the effect of violacein obtained from *C*. *violaceum* on different immune-related cell lines. We found that treatment with violacein was able to induce TNF-α expression in Raw 264.7 and ANA-1 cells. In addition, we determined the gene expression profile of Raw 264.7 cells incubated with this substance. Our results indicate the induction of inflammatory cytokines and suggest the activation of the TLR signaling pathway and in consequence an induction of inflammatory cytokines and negative regulators of TLR signaling. Using TLR-transfected HEK-293 cells, we determined that violacein significantly activates hTLR8 at 15 μmol/L or higher. Moreover, we found that the immunostimulatory activity of violacein in PBMCs was suppressed by the specific and potent hTLR8 antagonist, CU-CPT9a. Finally, we studied the interaction of violacein with hTLR8 *in silico* through molecular docking and obtained evidence that this binding could occur in a similar fashion to synthetic agonists of hTLR8. Our results show that violacein presents an immune stimulating effect in some murine cell lines and in PBMCs, and that this effect could be associated with signaling through TLR8.

## Results

### Purity of violacein

After purification, violacein was identified by comparison of ^1^H-NMR signals with previously reported data^19^. Additional signals were observed in the spectrum at very low intensity compared to violacein and they were consistent with common fatty acid impurities (δ 0.90, *t*, CH_3_ and δ 1.29, *br s*, (CH_2_)_n_)^20^. Characteristic signals related with Lipopolysaccharide contamination^21,22^ were not detected. Both HPLC-UV and ^1^H-NMR confirmed that violacein was obtained at highly purity (90–93%, measured by HPLC-UV).

### Effect of violacein on different murine cell types

The effect of violacein on cell viability was evaluated in the Raw 264.7 and ANA-1 cell lines, as well as in murine bone marrow-derived macrophages (BMM), plasmacytoid dendritic cells (pDC) and myeloid dendritic cells (mDC) from wild-type (wt), and from TLR7^−/−^ and TLR2/4^−/−^ knock-out mice.

**Figure 2:**
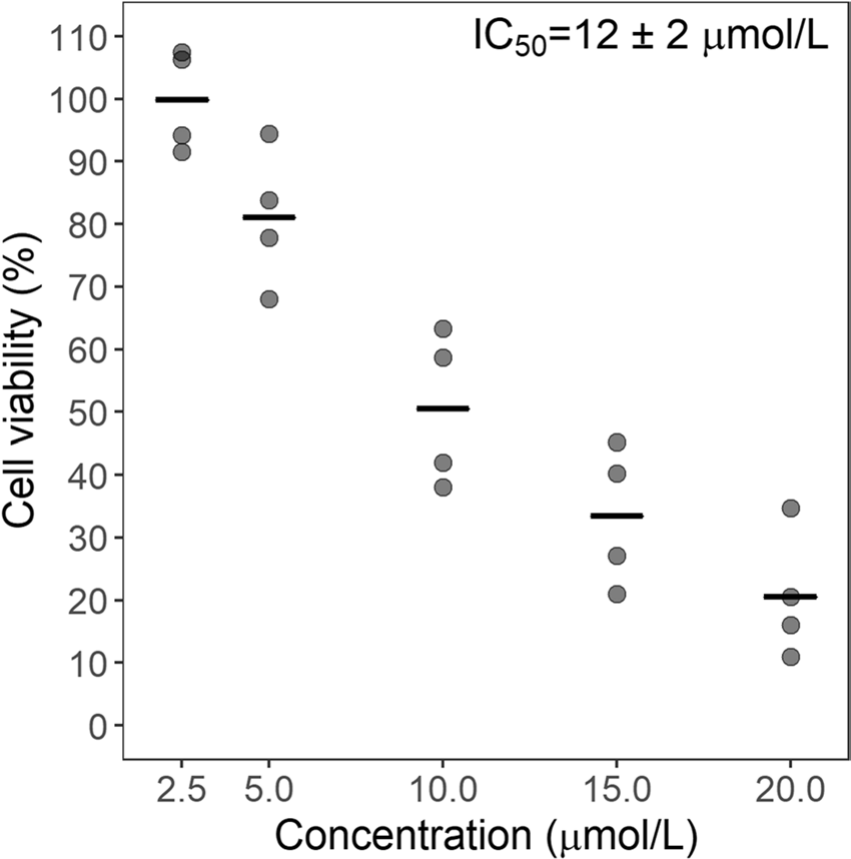
Cytotoxic effect of violacein on Raw 264.7 cells. Cells were incubated with the indicated concentrations of violacein for 24 h, and cell viability was evaluated using the MTT assay. The experiment was performed in a 96-well plate. Cell viability was defined as the percent ratio of absorbance in treated cells and that of control (amount of reduced MTT observed in the absence of compounds). Each data point represents one independent experiment run in triplicate and the center line indicates the mean.

To determine the effect of violacein on the Raw 264.7 cell line, cells were treated with different concentrations of the compound and cell viability was evaluated using the MTT assay. A cytotoxic effect of violacein was observed after 24 hours, with an IC_50_ value of 12 ± 2 µmol/L (mean ± standard deviation, n = 4, Fig. 2).

The effect of violacein on macrophage activation was initially determined by measuring nitric oxide production with the Griess reagent^23^. In macrophages, nitric oxide is produced by the inducible nitric oxide synthase, after cell stimulation. Raw 264.7 cells were incubated with increasing sub-toxic concentrations of violacein (1–12 µmol/L). As a positive control, lipopolysaccharide (LPS) from *E*. *coli* was added to a final concentration of 1.1 µg/mL. After 24 hours, 100 µl of supernatant were harvested and the production of nitric oxide was measured (data not shown). In contrast to LPS, treatment of cells with violacein did not produce a significant change in the nitric oxide levels when compared to unstimulated cells.

**Figure 3:**
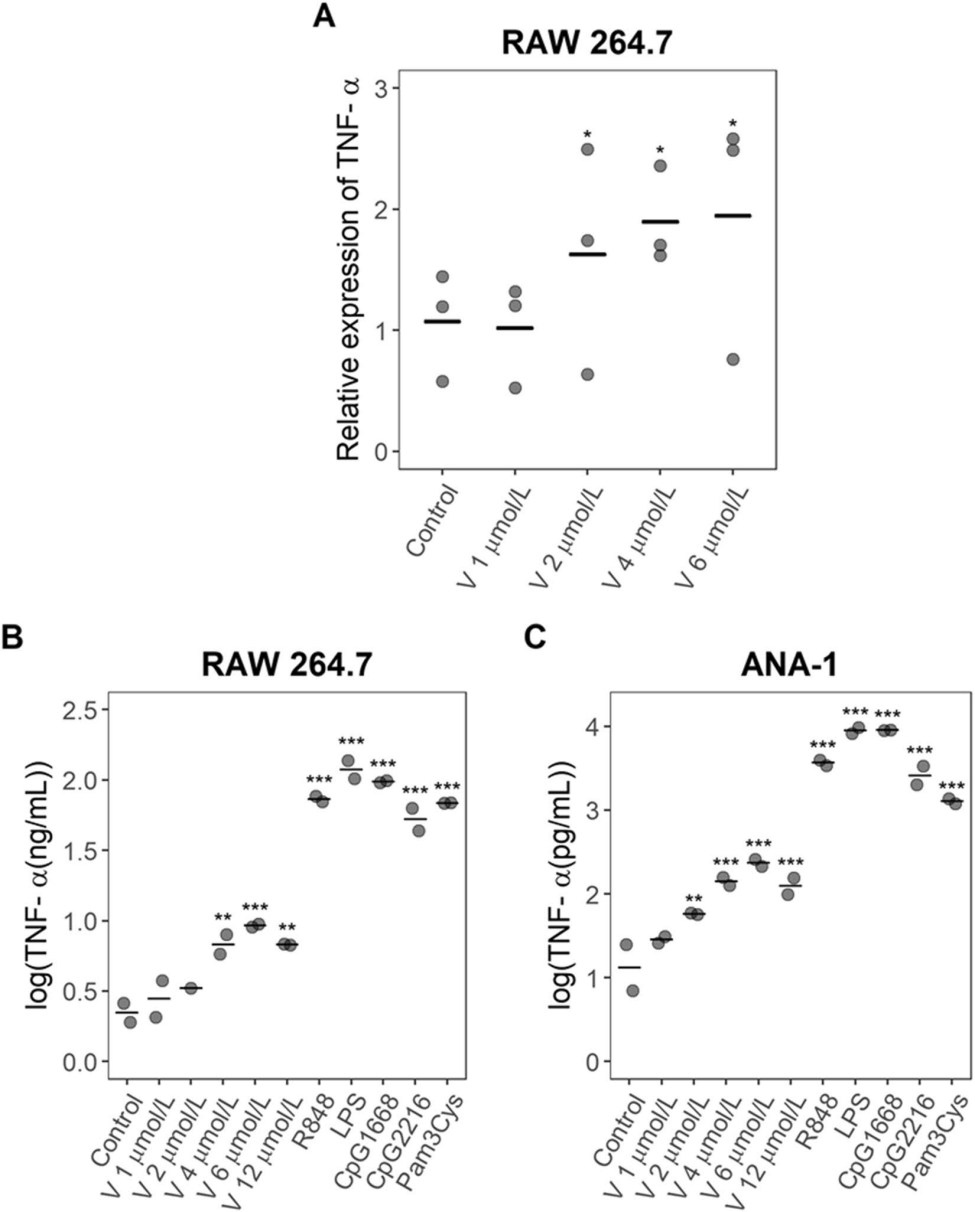
Effect of violacein on TNF-α production in Raw 264.7 and ANA-1 cells. (**A**) Raw 264.7 cells were treated with the indicated concentrations of violacein (V) and TNF-α was determined in total RNA extracts by real-time qRT-PCR. Relative TNF-α expression using GAPDH as a reference gene is shown. (**B)** Raw 264.7 cells or (**C**) ANA-1 cells were treated with the indicated concentration of violacein or incubated with various stimuli and TNF-α production was determined by ELISA. Each data point represents one experiment run in duplicate and the center line indicates the mean. **p < 0.01 compared to the untreated control, ***p < 0.001 compared to the untreated control.

Raw 264.7 cell activation by violacein was then investigated by measuring TNF-α expression using real-time qRT-PCR. Cells were treated with different violacein concentrations, and total RNA extracts were obtained after 4 hours. As shown in Fig. 3A, violacein at a 2 µmol/L or higher induces the expression of TNF-α mRNA in Raw 264.7 cells (stimulation index = 1.5). TNF-α production induced by violacein in Raw 264.7 and ANA-1 cells was also measured by ELISA, and consistent results were obtained (Fig. 3B,C): TNF-α production was increased at concentrations higher than 4 µmol/L in Raw 264.7 cells (SI = 3.1) or 2 µmol/L in ANA-1 cells (SI = 3.6), and a cytotoxic effect was observed for ANA-1 cells at 12 µmol/L.

To study the effect of violacein on primary murine cells, BMM, mDC and pDC from wt, TLR7^−/−^ and TLR2/4^−/−^ mice were incubated with violacein and TNF-α, IL-6 and IFN-α (only for pDC) production was measured by ELISA (data not shown). Production of the investigated cytokines was not induced, and we observed cytotoxicity at a concentration of 12 μmol/L (Table 1).

**Table 1:** Activation of different cell lines by violacein.

Cells	Activation
Raw 264.7	≥2 μmol/L^a^ and ≥4 μmol/L^b^
ANA-1	4 and 6 μmol/L^b^
PBMC	15 μmol/L^c^
Murine BMM (wt; TLR7^-/-^; TLR2/4^-/-^)	N.D.^d,e^
Murine mDC (wt; TLR7^-/-^; TLR2/4^-/-^)	N.D.^d,e^
Murine pDC (wt; TLR7^-/-^; TLR2/4^-/-^)	N.D.^d,e^
MonoMac 6	N.D.^d,f^
THP-1	N.D.^d,f^

^a^Data of real time qRT-PCR experiment for TNF-α.

^b^Data of ELISA experiment for TNF-α.

^c^Data of ELISA experiment for IL-6.

^d^N.D. = Not detected.

^e^Cell death was observed at 6 and 12 μmol/L.

^f^No cell death was observed at 12 μmol/L.

### Gene expression pattern induced by violacein in Raw 264.7 cells

Microarray analysis was performed for total RNA extracts obtained from Raw 264.7 cells that were cultured for 4 hours in the presence or absence of violacein (4 µmol/L). As shown in Table 2, of 41.346 genes analyzed, 129 showed a differential expression (≥1.5-fold change and LPE p-value < 0.05) when violacein-treated cells were compared to untreated cells. Of these, 46.5% of genes (60/129) showed an increase in expression, and 53.5% of genes (69/129) showed a decrease in expression. 26.4% of genes (34/129) were orphan genes and 9.3% of genes (12/129) corresponded to non-coding RNA.

**Table 2:** Differentially expressed genes in Raw 264.7 cells treated for 4 hours with 4 μmol/L of violacein, compared to untreated control cells.

Gene Symbol	Gene name	mRNA Accession	Fold change	p-value^a^
**UP-REGULATED GENES**				
Egr1	Early growth response 1	NM_007913	3.92	<1.52E-26
Plaur	Plasminogen activator, urokinase receptor	ENSMUST00000002284	2.79	<1.52E-26
Irg1	Immunoresponsive gene 1	ENSMUST00000022722	2.64	<1.52E-26
Gprc5a	G protein-coupled receptor, family C, group 5, member A	NM_181444	2.58	2.95E-11
Plk2	Polo-likekinase 2	NM_152804	2.55	<1.52E-26
Osm	Oncostatin M	ENSMUST00000075221	2.54	1.87E-11
Ccdc85b	Coiled-coil domain containing 85B	NM_198616//NM_198616	2.48	<1.52E-26
Tnf	Tumor necrosis factor	NM_013693	2.46	<1.52E-26
Il7r	Interleukin 7 receptor	NM_008372	2.46	3.18E-12
Pmepa1	Prostate transmembrane protein, Androgen induced 1	NM_022995	2.35	<1.52E-26
Rcan1	Regulator of calcineurin 1	NM_019466	2.28	<1.52E-26
Ccl2	Chemokine (C-C motif) ligand 2	NM_011333	2.27	8.48E-11
Cxcl2	Chemokine (C-X-C motif) ligand 2	ENSMUST00000075433	2.27	6.65E-07
Rgs16	Regulator of G-protein signaling 16	NM_011267	2.19	<1.52E-26
Plk3	Polo-like kinase 3	NM_013807	2.1	2.58E-08
Serpine1	Serine (or cysteine) peptidase inhibitor, clade E, member 1	NM_008871	1.99	2.22E-07
Rhob	Ras homolog gene family, member B	NM_007483	1.97	1.18E-05
Pdgfb	Platelet derived growth factor, B polypeptide	NM_011057	1.96	<1.52E-26
Gpr84	G protein-coupled receptor 84	ENSMUST00000079824	1.94	<1.52E-26
Traf1	TNF receptor-associated factor 1	NM_009421	1.93	9.26E-08
Dusp5	Dual specificityphosphatase 5	ENSMUST00000038287	1.92	1.79E-05
Itga5	Integrinalpha 5 (fibronectin receptor alpha)	NM_010577	1.9	<1.52E-26
Egr2	Earlygrowth response 2	NM_010118	1.9	2.74E-09
Dusp1	Dual specificity phosphatase 1	ENSMUST00000025025	1.88	<1.52E-26
Rgs1	Regulator of G-protein signaling 1	ENSMUST00000172388	1.88	3.90E-08
Slc6a8	Solute carrier family 6 (neurotransmitter transporter, creatine), member 8	NM_133987	1.82	<1.52E-26
Slc20a1	Solutecarrierfamily 20, member 1	NM_015747	1.79	<1.52E-26
Cxcl10	Chemokine (C-X-C motif) ligand 10	NM_021274	1.77	4.22E-05
**UP-REGULATED GENES**				
Egr3	Earlygrowth response 3	NM_018781	1.76	0.001266
Tm4sf19	Transmembrane 4 L six family member 19	NM_001160402	1.76	9.76E-09
Trib1	Tribbleshomolog 1 (Drosophila)	ENSMUST00000067543	1.74	0.000143
Tmem26	Transmembraneprotein 26	NM_177794	1.74	5.44E-09
Ier3	Immediateearly response 3	NM_133662	1.73	2.72E-10
Ptgs2	Prostaglandin-endoperoxidesynthase 2	NM_011198	1.71	3.14E-08
Nfkbia	Nuclear factor of kappa light polypeptide gene enhancer in B cells inhibitor, alpha	NM_010907	1.71	4.76E-12
Kdm6b	KDM1 lysine (K)-specific demethylase 6B	NM_001017426	1.7	4.11E-06
Myc	Myelocytomatosisoncogene	ENSMUST00000160009	1.69	0.000582
Skil	SKI-like	NM_011386	1.68	1.21E-06
Clec4e	C-type lectin domain family 4, member e	NM_019948	1.67	2.07E-11
Dusp4	Dual specificityphosphatase 4	ENSMUST00000033930	1.66	4.84E-08
Ccl4	Chemokine (C-C motif) ligand 4	NM_013652	1.66	4.94E-08
Zfp36	Zinc finger protein 36	ENSMUST00000051241	1.66	1.10E-09
Vegfc	Vascular endothelial growth factor C	NM_009506	1.66	0.003692
Nfkbie	Nuclear factor of kappa light polypeptide gene enhancer in B cells inhibitor, epsilon	NM_008690	1.62	0.00102
Tfrc	Transferrin receptor	NM_011638	1.61	1.37E-09
Ehd1	EH-domaincontaining 1	ENSMUST00000025684	1.61	8.10E-08
Tnfaip3	Tumor necrosis factor, alpha-inducedprotein 3	NM_009397	1.59	0.02444
Tgm2	Transglutaminase 2, C polypeptide	NM_009373	1.58	0.000104
Nfkb2	Nuclear factor of kappa light polypeptide gene enhancer in B cells 2, p49/p100	NM_001177369	1.58	4.68E-07
Nr4a1	Nuclear receptor subfamily 4, group A, member 1	NM_010444	1.55	0.001768
Junb	Jun-B oncogene	NM_008416	1.55	4.67E-08
Csrnp1	Cysteine-serine-rich nuclear protein 1	NM_153287	1.54	0.046319
Lims2	LIM and senescent cell antigen like domains 2	NM_144862	1.53	0.022744
Fos	FBJ osteosarcomaoncogene	NM_010234	1.53	5.79E-05
Rai14	Retinoicacidinduced 14	NM_030690	1.52	2.91E-05
Map2k3	mitogen-activated protein kinase kinase 3	NM_008928	1.52	1.36E-05
Fam129b	Family with sequence similarity 129, member B	NM_146119	1.51	5.29E-06
Plau	Plasminogenactivator, urokinase	NM_008873	1.51	3.01E-06
**DOWN-REGULATED GENES**				
Ccl3	Chemokine (C-C motif) ligand 3	NM_011337	1.51	8.85E-12
Rn5s20	5 S RNA 20	NR_046144//NR_046144	−1.87	1.52E-26
Adm	Adrenomedullin	NM_009627	−1.87	0.003717
Bex6	Brain expressed gene 6	NM_001033539	−1.82	0.046036
Gm5431	Predicted gene 5431	ENSMUST00000109212	−1.79	0.010583
S1pr1	Sphingosine-1-phosphate receptor 1	NM_007901	−1.79	0.000124
Tlr8	Toll-like receptor 8	ENSMUST00000112170	−1.77	0.004444
Rps20	Ribosomal protein S20	ENSMUST00000130128	−1.75	0.003227
Gm5771	Predicted gene 5771	NM_001038997	−1.74	0.027193
Trp53inp1	Transformation related protein 53 inducible nuclear protein 1	NM_001199105	−1.74	0.000304
Mxd4	Max dimerization protein 4	ENSMUST00000042701	−1.69	0.003729
Ccng2	Cyclin G2	ENSMUST00000121127	−1.68	0.005001
Snord58b	Small nucleolar RNA, C/D box 58B	NR_028552	−1.68	1.15E-11
Scel	Sciellin	NM_022886	−1.67	0.000661
Klhl24	Kelch-like 24 (Drosophila)	NM_029436	−1.66	7.08E-09
Gm7429//Gm6109//Rpl30	Predicted pseudogene 7429//predicted gene 6109//ribosomal protein L30	ENSMUST00000135417	−1.64	0.000191
Lrp2bp	Lrp2 bindingprotein	ENSMUST00000066451	−1.62	0.002288
Olfr820	Olfactory receptor 820	ENSMUST00000059244	−1.58	0.00979
Fbxl20	F-box and leucine-rich repeat protein 20	NM_028149	−1.58	0.016514
Ighm	Immunoglobulin heavy constant mu	AB067787//AB067787	−1.57	3.14E-08
9930111J21Rik2	RIKEN cDNA 9930111J21 gene 2//RIKEN cDNA 9930111J21 gene 2	BC066104//BC066104	−1.56	0.000145
Rny3	RNA, Y3 small cytoplasmic (associated with Ro protein)	NR_024202//NR_024202	−1.56	0.007455
Cysltr1	Cysteinylleukotriene receptor 1	ENSMUST00000113480	−1.54	0.00261
Bnip3	BCL2/adenovirus E1B interactingprotein 3	NM_009760	−1.54	5.28E-05
Clec7a	C-type lectin domain family 7, member a	NM_020008	−1.53	4.12E-06
Snord1b	Small nucleolar RNA, C/D box 1B	NR_028567	−1.52	9.76E-09
Dpep2	Dipeptidase 2	ENSMUST00000150001	−1.52	0.000407

^a^LPE p-value < 0.05 is considered significant.

To confirm the gene expression profile obtained with the microarray analysis, four genes were chosen (TNF-α, IRG1, CCL2 and CXCL2) for confirmation with real-time qRT-PCR. Selected genes showed >2.0-fold change in the microarray experiment. As shown in Table 3, all four genes showed an increase in expression in both microarray and real time PCR, and in both cases, up-regulation was highly significant as compared to untreated control cells.

**Table 3:** Confirmation of microarray results by comparison with real-time qRT-PCR for selected differentially expressed genes.

Gene name	Gene symbol	mRNA Accession	Microarray		Real time PCR	
			FC^a^	p-value^b^	FC	p-value^b^
Tumor necrosis factor alpha	TNF-α	NM_013693	2.46	<1.52E-26	5.19	0.003^c^
Immune responsive gene 1	IRG1	NM_008392	2.64	<1.52E-26	5.84	0.019^d^
Chemokine (C-C motif) ligand 2	CCL2	NM_011333	2.27	8.48E-11	6.52	0.002^e^
Chemokine (C-X-C motif) ligand 2	CXCL2	NM_009140	2.27	6.65E-07	10.53	1.87E-05^c^

^a^FC = Fold change.

^b^p < 0.05 is considered significant.

^c^n = 6.

^d^n = 4.

^e^n = 5.

The list of genes whose expression significantly changed after incubating Raw 264.7 cells with violacein was analyzed using the DAVID database, to understand the effect of violacein on biological pathways. Table 4 shows a list of the identified differentially expressed genes grouped by functional annotation. According to these results, violacein induced changes in expression of genes in biological pathways related with an immune and inflammatory response, apoptotic pathway and regulation of cell proliferation.

**Table 4:** Biological terms significantly associated with differential gene expression.

Term	Gene count	p-value^a^	Genes^b^
**SP_PIR_KEYWORDS**			
Inflammatory response	8	3.28E-08	CCL3, CCL2, CXCL2, **CLEC7A**, CCL4, KDM6B, **TLR8**, CXCL10
Cytokine	7	9.24E-05	OSM, CCL3, TNF, CCL2, CXCL2, CCL4, CXCL10
Chemotaxis	5	1.26E-04	CCL3, CCL2, CXCL2, CCL4, CXCL10
**GOTERM_BP_FAT**			
GO:0006954~inflammatory response	10	1.30E-06	CCL3, TNF, CCL2, MAP2K3, CXCL2, **CLEC7A**, CCL4, KDM6B, **TLR8**, CXCL10
GO:0006955~immune response	13	2.35E-06	CCL3, TNF, CCL2, CXCL2, **BNIP3**, NFKB2, IL7R, CCL4, **TLR8**, CXCL10, OSM, CLEC4E, **CLEC7A**
GO:0009611~response to wounding	11	6.13E-06	CCL3, TNF, CCL2, MAP2K3, CXCL2, **CLEC7A**, CCL4, KDM6B, **TLR8**, PLAUR, CXCL10
GO:0042127~regulation of cell proliferation	13	9.16E-06	TNF, CCL2, PTGS2, PDGFB, NFKBIA, CXCL10, VEGFC, **S1PR1**, **ADM**, SERPINE1, TGM2, MYC, PLAU
GO:0006952~defense response	11	5.54E-05	CCL3, TNF, CCL2, MAP2K3, CXCL2, **BNIP3**, **CLEC7A**, CCL4, KDM6B, **TLR8**, CXCL10
GO:0008284~positive regulation of cell proliferation	9	6.63E-05	VEGFC, TNF, **S1PR1**, CCL2, PDGFB, **ADM**, TGM2, MYC, CXCL10
GO:0006935~chemotaxis	6	1.83E-04	CCL3, CCL2, **CYSLTR1**, CXCL2, CCL4, CXCL10
GO:0045944~positive regulation of transcription from RNA polymerase II promoter	9	3.25E-04	OSM, EGR1, FOS, TNF, EGR2, **S1PR1**, CSRNP1, NR4A1, MYC
GO:0006917~induction of apoptosis	6	1.29E-03	TNF, TGM2, **TRP53INP1**, NR4A1, **BNIP3**, MYC
**GOTERM_MF_FAT**			
GO:0008009~chemokine activity	5	2.47E-05	CCL3, CCL2, CXCL2, CCL4, CXCL10
**KEGG_PATHWAY**			
mmu04620: Toll-like receptor signaling pathway	8	2.99E-06	FOS, CCL3, TNF, MAP2K3, NFKBIA, CCL4, **TLR8**, CXCL10
mmu04010: MAPK signaling pathway	11	5.59E-06	DUSP5, FOS, DUSP4, TNF, TM4SF19, PDGFB, DUSP1, MAP2K3, NR4A1, NFKB2, MYC
mmu04060: Cytokine-cytokine receptor interaction	10	2.17E-05	OSM, VEGFC, CCL3, TNF, CCL2, PDGFB, CXCL2, IL7R, CCL4, CXCL10
mmu04621: NOD-like receptor signaling pathway	5	6.93E-04	TNF, CCL2, CXCL2, NFKBIA, TNFAIP3

^a^Modified Fisher exact P-value, EASE Score; p < 0.05 is considered significant.

^b^Down-regulated genes are written in bold.

These results suggest that in murine cells, violacein activates Toll like receptor signaling (8 genes involved) and NOD-like receptor (5 genes involved) pathways.

### Effect of violacein on different human cell lines and PBMCs

To study the effect of violacein on human cells, TNF-α and IL-6 production by MonoMac 6, undifferentiated THP-1 cells and PBMCs was measured using ELISA. Neither cell type showed an induction of cytokine production at violacein concentrations up to 12 μmol/L . For this reason, TNF-α and IL-6 production was evaluated at higher concentrations with PBMCs from two donors. In this case, an induction of IL-6 production but not of TNF-α was observed (Table 1).

### Effect of violacein on HEK-293 cells transfected with hTLR8 or hTLR7

**Figure 4:**
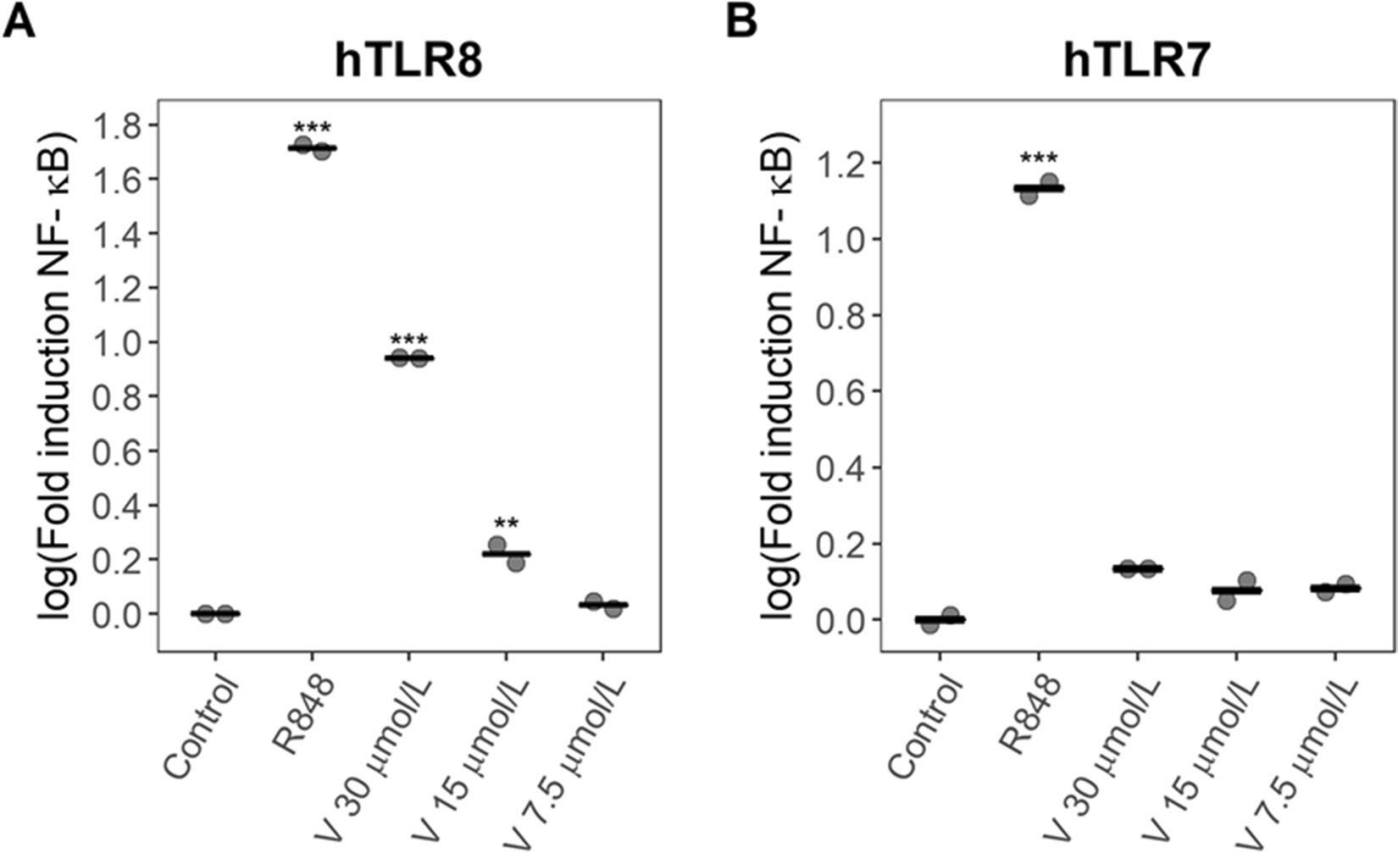
Effect of violacein on the induction of NF-κB in TLR-transfected HEK-293 cells with a NF-κB-luciferase reporter plasmid. (**A)** hTLR7 transfected HEK-293 cells were treated with indicated concentrations of violacein (V) and induction of NF-κB was determined by luciferase activity. (**B)** hTLR8 transfected HEK-293 cells were treated with indicated concentrations of violacein and induction of NF-κB was determined by luciferase activity. Each data point represents one replicate and the center line indicates the mean. **p < 0.01 compared to the untreated control, ***p < 0.001 compared to the untreated control.

To analyze the possibility that violacein could act as a hTLR7 or hTLR8 agonist, we measured NF-κB activation in HEK-293 cells expressing either hTLR7 or hTLR8 and a NF-κB-luciferase reporter plasmid. We observed that at 15 and 30 μmol/L, violacein caused a significant NF-κB induction (1.7 and 8.7-fold respectively) in hTLR8 transfected HEK-293 cells. Cell death was also observed at the highest concentration (Fig. 4A). In contrast, highest concentration of violacein caused a cytotoxic effect with no NF-κB induction in hTLR7 transfected HEK-293 cells (Fig. 4B). R848 (both TLR7 and TLR8 agonist) was used as a positive control. These results suggest that violacein can act as an agonist for hTLR8 and not for hTLR7.

### Blockade of violacein effect on PBMCs by a specific hTLR8 antagonist

**Figure 5:**
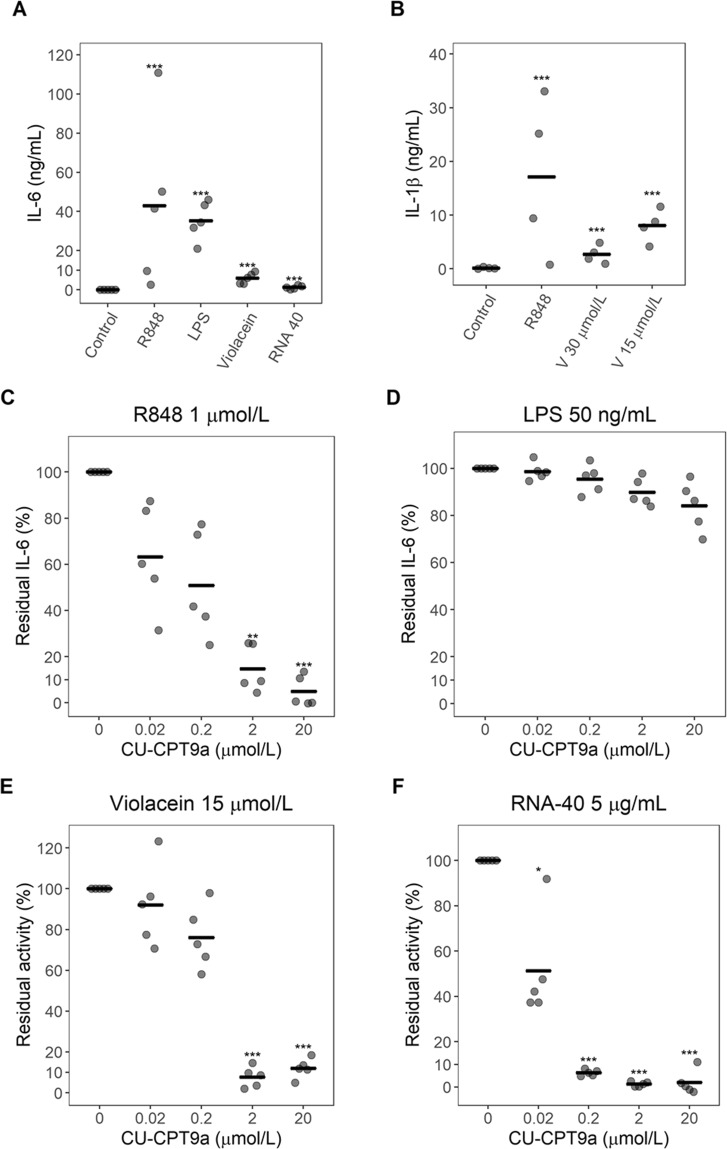
Effect of violacein and its antagonist on PBMCs (**A**). PBMCs from five donors were treated with 15 µmol/L violacein and the indicated controls and IL-6 production was determined by ELISA. (**B)** PBMCs from four donors were treated with 30 or 15 µmol/L violacein (V) or 1 µmol/L R848 and IL-1β production was determined by ELISA. (**C–F)**. PBMCs were treated with indicated concentrations of CU-CPT9a and then stimulated with 1 µmol/L R848, 50 ng/mL LPS, 15 µmol/L violacein or 5 µg/mL RNA-40. Cell activation was evaluated by the production of IL-6. Residual IL-6 percentage was defined as the percent ratio of IL-6 in cells treated with the antagonist and the stimulator compared to control cells (amount of IL-6 production observed in the absence of the antagonist). Each data point represents an individual donor (n = 5A, C, D, E and F; n = 4 B), the center line indicates the mean. *p < 0.05 compared to the untreated control, **p < 0.01 compared to the untreated control, ***p < 0.001 compared to the untreated control.

To explore the possibility that violacein acts via hTLR8, we investigated the effect of CU-CPT9a (4-(7-methoxyquinolin-4-yl)-2-methylphenol), a specific hTLR8 antagonist^24,25^, on its immunostimulatory effect on PBMCs. In the absence of the antagonist, we observed that at 30 μmol/L of violacein, PBMC viability was very low (13 ± 4%; mean ± standard deviation, n = 3) and no IL-6 production was detected. However, at 15 μmol/L viability was higher (48 ± 16%; mean ± standard deviation, n = 3) and there was a significant increase in IL-6 production in comparison to the untreated control (Fig. 5A). We also observed a significant production of IL-6 in presence of other stimuli, namely R848 or RNA-40 (TLR7/8 agonist) and LPS (TLR4 agonist) (Fig. 5A).

In order to assess the effect of CU-CPT9a on cell activation, PBMCs from five donors were incubated with different concentrations of CU-CPT9a (0, 0.02, 0.2, 2, 20 μmol/L), before addition of R848 (1 µmol/L), LPS (50 ng/mL), violacein (15 µmol/L) or RNA-40 (5 µg/mL).

PBMC treatment with R848 or RNA-40 induced IL-6 production, and this activation was suppressed by CU-CPT9a in a dose-dependent manner (Fig. 5C,F, respectively). In the case of R848, this suppression was significant at 2 and 20 μmol/L, whereas for RNA 40, a reduction was observed at all used antagonist concentrations.

When PBMCs were stimulated with LPS, IL-6 induction was not suppressed at any concentration of the antagonist (Fig. 5D). These results are evidence for antagonist specificity.

A dose-dependent suppression of IL-6 production by CU-CPT9a was also observed when cells were stimulated with violacein. A significant reduction was observed at 2 and 20 μmol/L in comparison to the no antagonist control (Fig. 5E). Interestingly the cytotoxic effect of violacein was not abolished by the antagonist.

In addition, when using IL-1β, as a marker, cytokine secretion was stimulated by violacein (30 and 15 µmol/L) and R848 (1 µmol/L) (Fig. 5B). However, previous addition of CU-CPT9a only blocked IL-1β stimulated by R848 and had no effect on violacein-induced IL-1β stimulation (data not shown).

### Molecular docking of violacein on TLR8

**Figure 6:**
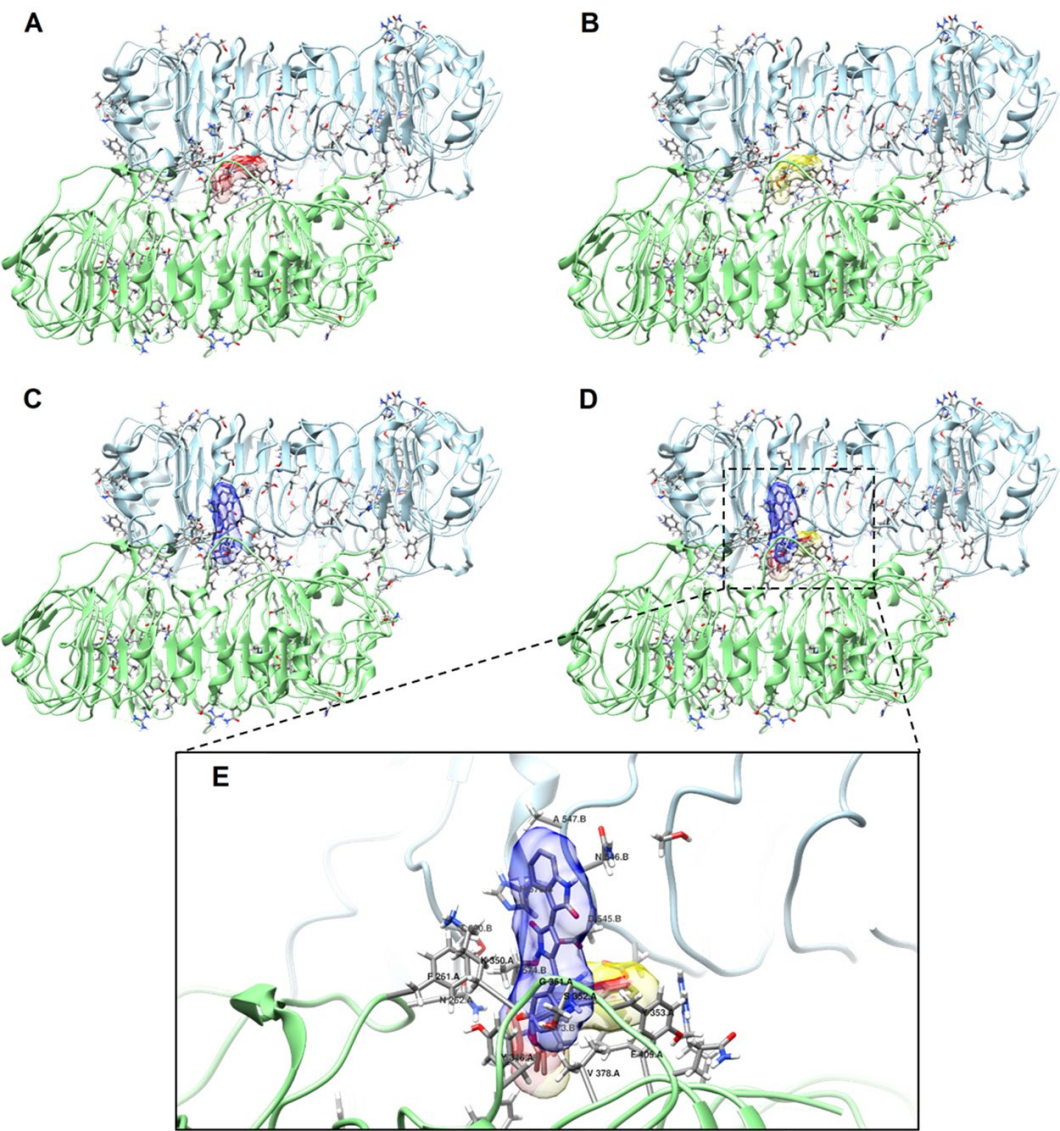
(**A**) Crystal structure of TLR8 bound to CL097 (PDB ID: 3W3J). (**B**–**E**) Docking results of ligand binding to hTLR8. (**B)** Docking of CL097 to hTLR8. (**C)** Docking of violacein to hTLR8. (**D)** Overlay of (**A)** (red), (**B)** (yellow) and (**C)** (blue). (**E)** Close-up view of overlay in (**D)**.

The molecular docking of hTLR8 with violacein or CL097, a synthetic hTLR8 agonist^26,27^, was performed using AutoDock Vina. Different binding models were obtained, and the one with the biggest change in binding free energy was selected (Fig. 6B,C). This model was compared to the crystal structure of hTLR8 bound to CL097^28^ (Fig. 6A). The simulated docking models of CL097 and violacein with TLR8 (Fig. 6) show that violacein and CL097 share similar binding modes by means of interactions with amino acids within the dimer interface as observed in the crystal structure of hTLR8 bound to CL097 (Fig. 6D,E). If violacein can interact with amino acids that are at a distance of 5 Å or lower within the hTLR8-hTLR8* dimer interface, the following amino acids could be in contact with the ligand: F261, N262, Y348, G351, S352, Y353, V378, F405, D545*, N546*, A547*, G572*, V573*, T574* and H576*.

## Discussion

Violacein is a natural product derived from bacteria, for which an array of biological effects have been described in the literature, such as immunosuppressive, analgesic, antipyretic and anti-inflammatory^7^. Moreover, treatment with violacein has been reported to reduce acute and chronic inflammation through the stimulation of regulatory T cells^12^. Contradictory evidence has been reported on whether violacein induces or inhibits TNF-α expression: while an induction has been observed in cell culture (MCF-7 breast cancer cells^13^ and HL-60 cells^9^), a reduction was observed on gastric mucosa in violacein-treated rats in which gastric lesions were induced with indomethacin^10^. A reduction of TNF-α expression was also observed upon violacein treatment in lumbar spinal cords of the EAE mouse model^12^. In this study, we researched the effect of violacein in different cell lines and primary cultured immune cells (human and murine) and obtained evidence for a cell-type specific activation and for the molecular mechanism of this activation.

We first studied whether violacein induced Raw 264.7 cell activation by measuring nitric oxide production. Our results show that violacein did not induce iNOS expression in Raw 264.7 cells after 24 h incubation. These observations are in agreement with previous reports that found that violacein decreases iNOS activity in gastric ulcers induced by indomethacin in rats^10^ and also that violacein does not cause significant changes in gene expression of iNOS in mouse lumbar spinal cord^12^. However, these results do not clarify whether violacein exerts an effect on macrophages. For this reason, we studied if violacein induces the production of TNF-α, which is related with macrophage activation.

TNF-α is a cytokine whose up-regulation is related with inflammatory activity, immune response and macrophage activation^15,16^. In this study, we determined that violacein induced TNF-α expression at sub-toxic concentrations in Raw 264.7 cells, at the mRNA (Fig. 3A) and protein level (Fig. 3B). Induction of TNF-α by violacein has been previously described in HL-60 cells, where the evidence suggests that TNF-α activates TNFR1, mediating apoptosis in leukemia cells^9^. Violacein was also shown to induce TNF-α gene expression in human breast cancer cells^13^.

Violacein also induced a weak production of TNF-α in ANA-1 cells at concentrations higher than 2 μmol/L (Fig. 3C). ANA-1 cells and Raw 264.7 cells are murine macrophages, and both cells lines were established by retroviral infection (Abelson murine leukemia virus for Raw 264.7, J2 retrovirus for ANA-1)^29,30^.

Cytokine production (TNF-α, IL-6 and IFN-α only for pDC) in murine BBM, mDC and pDC from wild-type mice, or TLR7^−/−^ and TLR2/4^−/−^ knock-outs were not induced after treatment with violacein. Thus, our results indicate that violacein only presents an activity in virus-transformed murine macrophages such as Raw 264.7 and ANA-1 cells. In both cases, up-regulation of TNF-α but not IL-6 was observed.

Due to our observation of a TNF-α up-regulation by violacein in Raw 264.7 cells, we decided to study the gene expression profile in this cell line. According to our results (Table 4), the global changes in gene expression that were caused by incubation with violacein agree with an activation of macrophages. We observed differential expression of genes involved in an inflammatory response and signaling (TNF-α, MAP2K3, KDM6B, TLR8), immune response (BNIP3, NFKB2, IL7R, TLR8, OSM) and chemotaxis (CCL2, CCL3, CXCL2, CCL4, CXCL10). The expression of genes associated with regulation of cell proliferation (TNF-α, PTGS2, PDGFB, VEGFC) and apoptosis (TNF, TGM2, TRP53INP1, BNIP3, MYC) was also significantly affected. We also found an association with signaling pathways through PAMP-receptors such as Toll-like receptor (FOS, CCL3, TNF-α, MAP2K3, NFKBIA, CCL4, TLR8, CXCL10) and NOD-like receptor (TNF-α, CCL2, CXCL2, NFKBIA, TNFAIP3). These observations suggest that violacein could activate a Toll-like receptor in murine cells. Given that we found the expression of mTLR8 to be downregulated, we think that the effect of violacein in murine macrophages is related with this receptor.

TLR8 is involved in the recognition of single stranded RNA (ssRNA) and initiates an immune response that can signal via two distinct mechanisms involving different adapter proteins, namely MyD88 or TRIF. The MyD88-dependent pathway results in the activation of NF-κB and activated protein-1 (AP-1or FOS) transcription factors, while the TRIF-dependent pathway results in the activation of type I interferons^17,28^. hTLR8 can recognize single stranded RNA (ssRNA) and imizadoquinolines and induce an immune response. In contrast, mTLR8 is not activated by ssRNA or imizadoquinolines^31^. However, mTLR8 can be activated by imidazoquinolines in combination with polyT oligonucleotides, causing NF-κB activation and TNF-α expression in HEK-293 cells^31^. Moreover, mTLR8 overexpression can also induce NF-κB activation and TNF-α production but does not activate AP-1 and interferon-α^32^. These two studies show that mTLR8 is indeed functional.

Our data indicate that violacein activates hTLR8 and suggest that violacein could act via mTLR8 signaling in Raw 264.7 cells. Moreover, the gene expression results related with the activation of mTLR8 do not show differential expression of interferons, this observation is in agreement with previous report of activation of mTLR8^31^.

Expression of mTLR8 in the microarray data was found to be down regulated. This could be the consequence of a negative feedback mechanism to shut down TLR signaling. This possibility is supported by the observation that IκBα and IκBε were up-regulated. Both proteins are related with TLR signaling inhibition, specifically by inhibition of the transcription factor NF-κB. Furthermore, the expression of IRG1 leads to negative regulation of TLR-mediated signaling by stimulating A20 via the induction of reactive oxygen species production^33^. A20 is also involved in feedback inhibition of NF-κB activation^34^. Both genes (IRG1 and A20) were up-regulated upon violacein incubation, and the activation of IRG1 was also confirmed by real-time qRT-PCR.

When assessing the effect of violacein on cytokine expression in human cells, no induction was observed with THP-1 and MonoMac 6 human macrophage cell lines. PBMCs did not produce TNF-α or IL-6 at violacein concentrations of 12 μmol/L or lower. However, IL-6 expression was observed at higher violacein concentrations (Table 1, Fig. 5A). IL-6 is produced by several cell types such as macrophages, dendritic cells and B cells^35^. It is also involved in the control of T-helper differentiation, where it promotes Th2 differentiation and simultaneously inhibits Th1^36^.

Owing to the structural similarity between mTLR8 and hTLR8 and between hTLR8 and hTLR7, we decided to research the possibility that violacein could act as an agonist of hTLR8 or hTLR7. To do this, we studied the ability of violacein to induce NF-κB in hTLR7 or hTLR8 transfected HEK-293 cells. Our results indicate that violacein is acting through hTLR8, and not via hTLR7 (Fig. 4). Interestingly, NF-κB activation in hTLR8 transfected HEK-293 cells could only be observed at cytotoxic concentrations of violacein. According to this, cell death and activation via TLR8 are associated in HEK-293 cells.

To obtain further evidence that violacein is acting via hTLR8, we used CU-CPT9a, a known specific hTLR8 antagonist but not of hTLR7^24,25^. We observed that the immunostimulatory effect of violacein in PBMCs was abolished with the highest concentration of the antagonist and the same behavior was observed when the cells were stimulated with R848 or RNA-40 (TLR7 and TLR8 agonists) but not with LPS (TLR4 agonist). These results support the idea that violacein is activating hTLR8 directly or indirectly. Furthermore, we observed that PBMC treatment with violacein induced IL-1β secretion, which suggests the activation of inflammasome and pyroptosis^37,38^. In contrast to NF-κB activation, IL-1β expression was not blocked by CU-CPT9a.

Due to our observation that violacein can induce signaling through hTLR8, we decided to address the possibility that violacein could interact with hTLR8. To do this, we performed molecular docking calculations to simulate the interaction between violacein and hTLR8. Our results (Fig. 6) show that violacein could interact with hTLR8 in a similar manner to the imidazoquinoline CL097 (a derivative of resiquimod), based on the X-ray structure of hTLR8 bound to this synthetic agonist^28^. In the best binding model of violacein to hTLR8, this ligand presents a higher affinity (∆G = −9.4 kcal/mol) for the receptor than the best model obtained for CL097 (∆G = −8.8 kcal/mol). According to this model, violacein could interact with the following amino acids in the dimer interface of TLR8-TLR8*: F261, N262, Y348, G351, S352, Y353, V378, F405, D545*, N546*, A547*, G572*, V573*, T574*, H576* and T600*. In comparison, CL097 was found to interact with twelve amino acids (F346, Y348, G376, V378, I403, F405, V520*, D543*, D545*, T574*, G572*, V573*)^28^. In summary, we found that violacein could interact with six amino acids that are also involved in ligand binding in the X-ray structure of CL097 bound to hTLR8. In detail, we observed the following interactions to be analogous to those found for CL097: a hydrogen bond between the amino group of the 2-pyrrolidone ring of violacein with Thr574*, and a hydrophobic interaction of the 5-hydroxyindole ring of violacein with a hydrophobic pocket (Y348, V378, F405, G572* and V573*). Furthermore, specific residues (F405, Y348, T574*, D545* and D543*) have shown to be important in the activation of a transduction pathway that leads to NF-κB activation^28^. In our molecular docking model, we observed an interaction of violacein with the majority (5 out of 6) of these residues.

Regarding the effect of violacein on murine cell lines and PBMCs, we observed that treatment leads to the stimulation of a pro-inflammatory response. In this sense, our results are in agreement with previous data obtained with other immune cell lines^9,13^. Moreover, our results also offer an explanation for the anti-inflammatory effect that is observed in the animal models^7,10,12^: according to our gene-expression results, incubation with violacein could lead to an induction of negative feedback of TLR signaling, and promotion of pro-apoptotic processes (TNF-α, TGM2, TRP53INP1, NR4A1, BNIP3, MYC). This is also in agreement with previous results that show that violacein induces programmed cell death^9,13,39–43^.

Apoptosis due to violacein has been studied previously in different cancer cell lines and there is no agreement on which cell death mechanism is activated by this substance^9,39–44^. These previous studies suggest that the programmed cell death activated by violacein is specific for the cancer cell lines. However, the study of the mechanism of programmed cell death in PBMCs and in immune cell lines used in this study is beyond the scope of this manuscript.

In conclusion, the current study found that violacein induces TNF-α activation at non-cytotoxic concentrations in two murine cell lines (Raw 264.7 and ANA-1) established by retroviral infection, and cell death was observed at the highest concentration tested (12 µmol/L). Activation of PBMCs by violacein was detected at higher concentrations than used for murine cells, and was abolished by CU-CPT9a, a specific hTLR8 antagonist.

Based on gene expression analysis, we found that violacein induces activation of biological processes such as an immune response, an inflammatory response, signaling through MAPK pathway, cytokine-cytokine receptor interaction and Toll-like receptor signaling in Raw 264.7 cells. Our results suggest that the observed response in PBMCs implies activation of hTLR8 signaling. Finally, according to *in silico* analysis, violacein could bind to hTLR8 in a similar fashion to imidazoquinoline compounds.

TLR8 agonists show promise in immune therapy. For example, resiquimod, has been useful in the treatment of skin cancer^45^, viral skin lesions^46^ or as a vaccine adjuvant^47,48^. For this reason, we propose that violacein could have potential contribution in future immunotherapy strategies.

## Methods

### Culture of *C*. *violaceum*

*C*. *violaceum* was grown aerobically in Erlenmeyer flasks containing 500 mL of LB medium at 25 °C and shaking. Cultures were inoculated at an initial optical density (OD_600_) of 0.05 and grown for 17 hours.

### Extraction, purification and characterization of violacein

The culture of *C*. *violaceum* (1 L) was centrifuged (4 000 rpm at 18 °C, 15 min) and the bacterial pellet was extracted with ethanol at room temperature for 1 h. Then, the extract was sonicated for 6 min in an ultrasonic bath, centrifuged to remove cellular debris and the supernatant dried to yield a crude extract. The crude extract was washed once with water and three times with hexane, followed by sonication in an ultrasonic bath. Violacein was separated from the crude extract by crystallization with MeOH:H_2_O (30:70), and a violet solid was obtained. Then, the solid was loaded on a solid phase extraction (SPE) column (C8), washed with a series of MeOH:H_2_O mixtures of increasing polarity, and the purple fractions were combined and dried. Finally, the purple solid was purified by high performance liquid chromatography (HPLC-UV) using the following conditions: room temperature; mobile phase: 15 min 35:65 H_2_O:MeOH, 2 min 100% methanol and 2 min 35:65 H_2_O:MeOH; detector wavelength: 230 nm; stationary phase: Phenomenex Luna column C18 (250 × 4.6 mm, 10 µm). The purple fractions for each injection were combined and the final solution was dried. Violacein yield was 0.6 ± 0.1 mg. Violacein was characterized by ^1^H-NMR and ^13^C-NMR and UV-Vis. The purity of violacein was determined by reverse-phase HPLC-UV using the following conditions: room temperature; mobile phase: 0–30 min 35:65 H_2_O:MeOH (isocratic); detector wavelength: 230 nm; stationary phase: Phenomenex Luna semi-preparative column C18 (250 × 4.6 mm, 10 µm). The purity of violacein was 91 ± 2%.

### Ethics statement

The use of anonymous blood samples for this study has been approved by the local ethic committees of the Justus-Liebig-University Giessen and Philipps-University Marburg. The human samples (buffy coats from blood donors) were provided by the Institute for Clinical Immunology and Transfusion Medicine, Justus-Liebig-University Giessen, Germany. We confirm that all methods for drawing blood and preparation of buffy coats were performed in accordance with local guidelines and regulations. We also confirm that blood products were obtained only after informed consent from the blood donors. Human peripheral blood mononuclear cells (PBMCs) were isolated from buffy coats by Ficoll density gradient centrifugation with LSM 1077 (PAA). The experiments with murine tissue were performed in accordance with the National German welfare law §4 (3) TierSchG and §2 and Annex 2 (TierSchVerV) of the National Order for the use of animals in research. They were approved by the Philipps-University Marburg and supervised by the corresponding animal welfare officer.

### Mice and cells

TLR7-deficient, TLR2/4-double deficient and C57BL/6 WT mice were kept under specific pathogen free (SPF) conditions in the animal facility of the Philipps-University of Marburg. Mouse bone marrow cells were differentiated into macrophages, myeloid or plasmacytoid DC. Primary macrophages and mDC were cultivated in RPMI supplemented with 10% FCS, 1% L-glutamine, penicillin and streptomycin, 0.1% mercaptoethanol and cultured with 20 ng/mL M-CSF (primary macrophages) or 20 ng/mL GM-CSF (mDC) in a 5% CO_2_ humidified atmosphere at 37 °C. pDC were cultured in Optimem supplemented with 1% FCS, 100 U/mL penicillin, 100 μg/mL streptomycin 0.05 mmol/L mercaptoethanol and with Flt-3 ligand in a 5% CO_2_ humidified atmosphere at 37 °C. Macrophages, mDC and pDC were seeded at 2 × 10^5^ cells/well.

Raw 264.7 and ANA-1 cells were cultured in DMEM medium supplemented with 10% heat-inactivated FBS, 1% penicillin and streptomycin in a 5% CO_2_ humidified atmosphere at 37 °C. Raw 264.7 and ANA-1 cells were seeded at 1 × 10^5^ cell/well.

MonoMac 6, non-differentiated THP-1 and PBMCs were cultured in RPMI medium supplemented with 10% inactivated FBS, 1% penicillin and streptomycin in a 5% CO_2_ humidified atmosphere at 37 °C and were seeded at 2 × 10^5^ cell/well.

### Cell stimulation

Violacein was dissolved in dimethyl sulfoxide (DMSO) at a final concentration of 2.9 mmol/L or 30 mmol/L. The desired concentration of violacein in the experiments was attained by diluting with culture media. For cell viability and cytokine induction experiments, cells were incubated for 16–20 h with positive controls or different concentrations of violacein.

### Cell viability

Cell viability was assessed using a MTT assay according to Mosmann, T.^49^, with modifications. Raw 264.7 cells were seeded in triplicate on a 96-well plate and incubated for 24 h at 37 °C, 5% CO_2_. Different concentrations of violacein were added and the cells were incubated for 24 h at 37 °C, 5% CO_2_ (Final volume = 200 μL). Subsequently, MTT was added to each well and the incubation was continued for 4 h at 37 °C. The media was discarded, and the formazan crystals were dissolved in acidified-isopropanol. Absorbance was read in a microplate reader (MRX revelation DYNEX Magellan Biosciences) at 570 nm with a reference wavelength of 630 nm.

### Cytokine measurement

Concentration of different human and murine cytokines (TNF-α, INF-α, IL-6 and IL-1β) in the culture supernatant were measured by ELISA according to the manufacturer’s instructions (R&D Biosystems for murine IL-6 and human 1L-1β, BD bioscience for murine and human TNF-α and Pharmingen for human IL-6). Murine INF-α was analyzed with PBL interferon source. Cytokine production significantly above untreated control was interpreted as an activation of gene expression.

### Real-time qRT-PCR

Real-time qRT-PCR was performed according to Sripanidkulchai, *et al*.^50^, with modifications. Raw 264.7 cells were cultured in DMEM as described above and seeded (500 μL of 2 × 10^6^ live cells/mL per well) on a 24 well plate and were incubated for 24 h at 37 °C, 5% CO_2_. After this, different concentrations of violacein were added and cells were incubated for another 4 h (Final volume = 1000 μL). Total RNA was extracted using RNeasy Mini kit (QIAGEN) following the manufacturer’s instructions. RNA quality was determined with the ratio of absorbance 260/280 nm in a NanoDrop spectrophotometer. Reverse transcription was performed using RevertAid RT Reverse Transcription kit (Life Technologies) following the manufacturer’s instructions. Quantitative real-time PCR was performed using an Applied Biosystems 7500 Real Time PCR system, using SYBR Green master mix (Applied Biosystems) following the manufacturer’s instructions. The temperature was 95 °C for 10 min, followed by 40 cycles of amplification (95 °C for 15 s, 60 °C for 60 s) followed by the measurement of a melting curve. The analyzed genes were TNF-α, CCL2, CXCL2, IRG1 and GAPDH as a reference gene. All primers were designed, and specific sequences and product sizes are summarized in Table 5. TNF-α (200 units per well) was used as a positive control and primers for TNF-α, CCL2, CXCL2, IRG1 were used for microarray validation. Table 5 presents the primers used in the real-time qRT-PCR.

**Table 5:** Gene-specific primers used for real-time PCR.

mRNA Accession	Gene symbol	Primer 5′ to 3′	Product size (bp)
NM_001289726.1	GAPDH	F: TGACGTGCCGCCTGGAGAAA	98
		R: AGTGTAGCCCAAGATGCCCTTCAG	
NM_013693	TNF-α	F: CGGGCAGGTCTACTTTGGAG	166
		R: ACCCTGAGCCATAATCCCCT	
NM_011333	Ccl2	F: CACTCACCTGCTGCTACTCA	117
		R: GCTTGGTGACAAAAACTACAGC	
NM_009140	Cxcl2	F: TGAACAAAGGCAAGGCTAACTG	118
		R: CAGGTACGATCCAGGCTTCC	
NM_008392	Irg1	F: CAACATGATGCTCAAGTCTGTC	101
		R: TCCTCTTGCTCCTCCGAATG	

### Gene expression profile

Raw 264.7 cells were seeded on a 24-well plate (1 × 10^6^ live cells/mL per well) and were incubated for 24 h. Violacein was then added at a final concentration of 4 µmol/L per well. For the negative control, the same volume of fresh medium was added. Cells were incubated for 4 h, and all conditions were setup in triplicate.

Total RNA was extracted using the RNeasy Mini kit for total RNA isolation (QIAGEN) as per the manufacturer’s instructions. RNA samples were sent to Macrogen Inc. (Seoul, South Korea) for carrying out the microarray experiment and analysis.

Genes with greater than or equal to 1.5-fold change and LPE (Local-pooled error) p-value less than 0.05 were considered significantly differentially expressed. A total of 129 genes were found to be differentially expressed. Functional and pathway analysis for genes with differential expression was performed using the Database for Annotation, Visualization and Integrated Discovery (DAVID)^51,52^. Pathway enrichment was determined by a Fisher exact test. A p-value of less than or equal to 0.05 and a minimum of 5 genes in the pathway were required to consider that this pathway is involved in the response to violacein. Gene expression data were deposited in NCBI’s Gene Expression Omnibus (GEO, http://www.ncbi.nlm.nih.gov/geo) with accession number GSE82136.

### TLR-transfected HEK-293 cell assay

HEK-293 cells stably expressing TLR7 or TLR8 (Invivogen, Toulouse, France) were additionally stably transfected with an NF-κB-luciferase reporter plasmid (pGL3-Gluc, Thomas Zillinger, University of Bonn, Germany) by cotransfection with the expression plasmid pMSCVpuro. After clonal expansion, clones were selected and tested. For violacein stimulation experiments, cells were seeded at 3 × 10^4^ live cell/well in 96 well plates, and incubated with different concentrations of violacein (0.9 to 30 μmol/L) or R848 as a positive control. After 24 h of stimulation, the supernatant was collected and discarded. 50 µL of Lysis Juice (PJK, Kleinblittersdorf, Germany) was added to each well and cells were lysed by freezing at −80 °C for at least 20 min. 10 µL of the lysate was mixed with 30 µL of Gaussia luciferase buffer (1.43 µmol/L Coelenterazin, 2.2 mmol/L Na_2_EDTA, 0.22 mol/L K_x_PO_4_, 0.44 mg/mL BSA, 1.1 mol/L NaCl, 1.3 mmol/L NaN_3_) and measured with a Berthold luminometer (Pforzheim, Germany). The n-fold induction was obtained by dividing the value of the stimulus by the media control.

### Synthesis of CU-CPT9a

**Figure 7:**
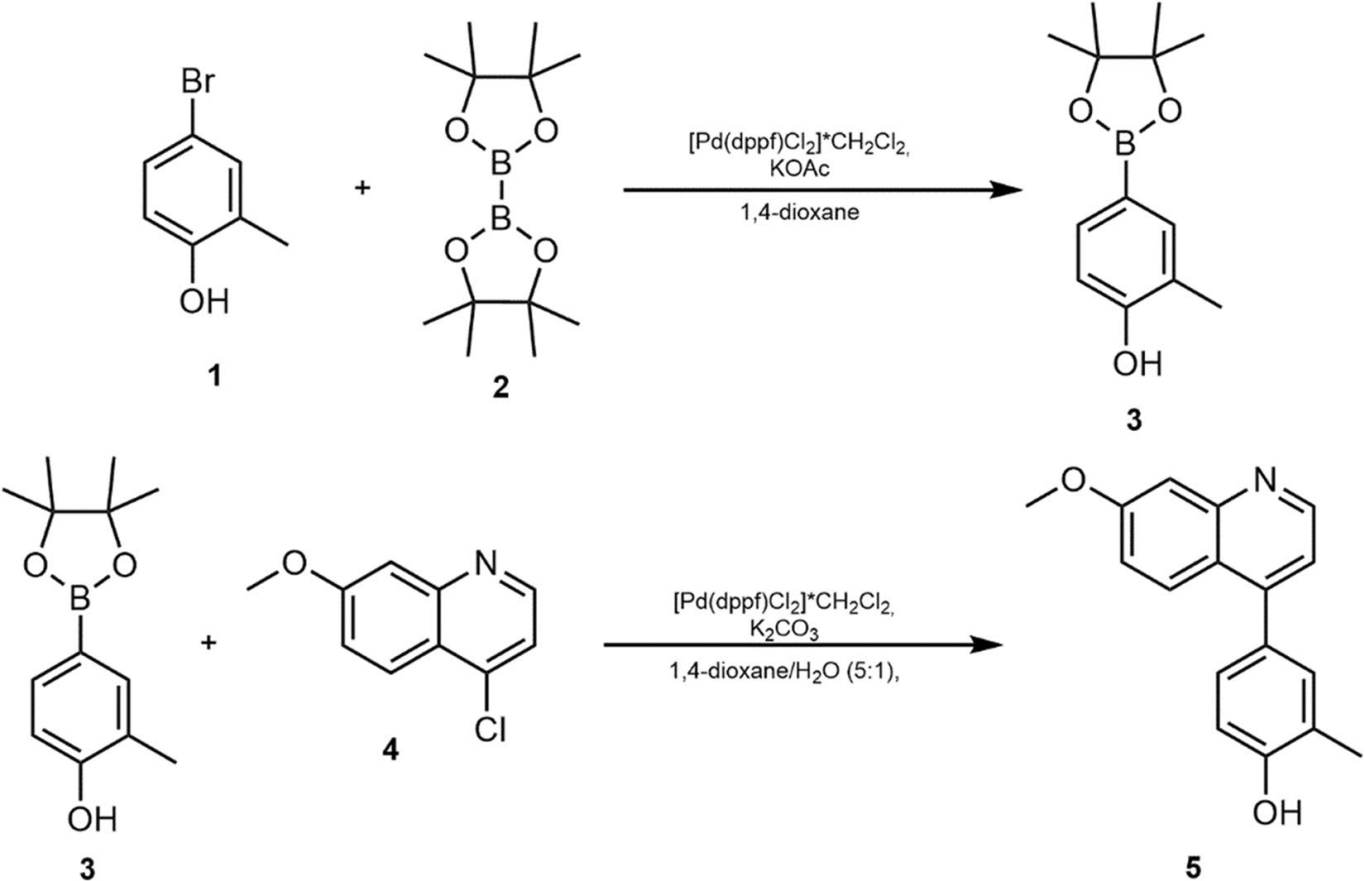
Synthesis of CU-CPT9a. Structures of 4-bromo-2-methylphenol (1), bis-(pinacolato)-diboron (2), 2-methyl-4-(4,4,5,5-tetramethyl-1,3,2-dioxaborolan-2-yl)-phenol (3), 4-chloro-7-methoxyquinoline (4) and 4-(7-methoxyquinolin-4-yl)-2-methylphenol (CU-CPT9a, 5).

General. All reagents and solvents were commercially available and used without further purification. All reactions were carried out under an argon atmosphere using Schlenck-technique. ^1^H-NMR spectra were recorded at 250 MHz on a Bruker Avance 250 spectrometer at 20 °C. Chemical shifts (δ) are given in ppm with the residual solvent signal used as reference. Coupling constants are reported in Hertz (Hz) using the following abbreviations for signal multiplicity: br (broad), s (singlet), d (doublet) and m (multiplet). Thin layer chromatography (TLC) was performed on precoated plates (silica gel 60 F254, Merck). Flash column chromatography was performed on prepacked columns (PF-30SIHP-JP/40 G; Interchim) using a Büchi separation system. Quantitative NMR (qNMR) measurements for compound CU-CPT9a (Fig. 7 compound 5) were recorded on a Jeol ECA-500 spectrometer using maleic acid, purchased from Sigma-Aldrich (99.94% purity), as internal reference standard.

2-Methyl-4-(4,4,5,5-tetramethyl-1,3,2-dioxaborolan-2-yl)-phenol (Fig. 7 compound 3) was prepared via a slightly modified literature procedure^24^ as follows: In a three-necked flask 5.88 g potassium acetate (60.0 mmol, 3.00 eq) were dried *in situ* (200 °C, 4 × 10^−3^ mbar) and suspended in anhydrous 1,4-dioxane (300 mL). 3.74 g 4-bromo-2-methylphenol (20.0 mmol, 1.00 eq), 6.10 g bis-(pinacolato)-diboron (24.0 mmol, 1.20 eq) and 820 mg [PdCl_2_(dppf)]*CH_2_Cl_2_ (1.00 mmol, 0.05 eq) were added and the orange-red suspension was stirred under argon atmosphere at 90 °C for 17 h. After cooling down to room temperature, 300 mL water were added and the suspension was extracted with ethyl acetate (3 × 150 mL). The combined organic layers were washed with brine, dried over magnesium sulfate, filtered and the solvent was removed under reduced pressure. The crude product was purified by flash column chromatography (silica gel, eluent: dichloromethane) to give 4.29 g of the title compound as a beige solid (18.3 mmol, 92% yield).

^1^H-NMR (250 MHz, CDCl_3_) δ 7.60 (s, 1H), 7.56 (d, *J* = 7.9 Hz, 1H), 6.76 (d, *J* = 7.9 Hz, 1H), 4.95 (s, br, 1H), 2.25 (s, 3H), 1.33 (s, 12H) ppm. All recorded spectra are in accordance to literature^24^.

4-(7-Methoxyquinolin-4-yl)-2-methylphenol (CU-CPT9a, Fig. 7 compound 5) was prepared via a slightly modified literature procedure^24^ as follows: In a nitrogen-flask 2.66 g 2-methyl-4-(4,4,5,5-tetramethyl-1,3,2-dioxaborolan-2-yl)-phenol (11.4 mmol, 1.10 eq), 2.00 g 4-chloro-7-methoxyquinoline (10.3 mmol, 1.00 eq) and 4.27 g potassium carbonate (30.9 mmol, 3.00 eq) were flushed with argon. The mixture was suspended in 1,4-dioxane (120 mL) and water (20 mL) and degassed. Afterwards, 425 mg [PdCl_2_(dppf)]*CH_2_Cl_2_ (0.52 mmol, 0.05 eq) were added and the orange-red suspension was heated at 100 °C until TLC indicated completion of the reaction (18 h). Subsequently, the mixture was concentrated to 50 mL under reduced pressure and filtered over Celite^®^. The filtrate was diluted with water (100 mL) and extracted with ethyl acetate (3 × 50 mL). The combined organic layers were dried over magnesium sulfate, filtered and the solvent was removed under reduced pressure. The residue was purified by flash column chromatography (silica gel, eluent: dichloromethane → dichloromethane/methanol 99:1) to give 2.17 g of the title compound as a white solid (8.16 mmol, 79% yield). Purity: 97.4 ± 0.3% (^1^H-qNMR). ^1^H-NMR (250 MHz, DMSO-d_6_) δ 9.69 (s, 1H), 8.79 (d, *J* = 4.6 Hz, 1H), 7.85 (d, *J* = 9.3 Hz, 1H), 7.44 (d, *J* = 2.3 Hz, 1 H), 7.25–7.20 (m, 3 H), 6.95 (d, *J* = 8.2 Hz, 1H), 3.92 (s, 3H), 2.21 (s, 3H) ppm. All recorded spectra are in accordance to literature^24^.

### Inhibition of hTLR8 activity by CU-CPT9a

Human PBMCs were cultured (50 µL of 6 × 10^6^ live cells/mL) in a 96-well plate. Cells were treated with different concentrations of CU-CPT9a (0.02, 0.2, 2 and 20 µmol/L) or medium as a negative control and were incubated for 1 h at 37 °C, 5% CO_2_. After this, cells were treated with violacein (30 and 15 µmol/L), R848 (1 µmol/L), LPS (50 ng/mL) or RNA-40 (5 µg/mL). After incubating for 20 h, the culture supernatants were collected and used to measure the concentration of IL-6 or IL-1β by ELISA according to the manufacturer’s instructions.

### Molecular docking

Molecular docking studies were performed using AutoDock Vina^53^. The three dimensional structure of hTLR8 complexed to 2-(ethoxymethyl)-1H-imidazo[4,5-c]quinolin-4-amine (CL097) was extracted from the Protein Data Bank (PDB ID 3W3J,^28^). The structure of the ligand was drawn with Marvin Sketch. The structures of ligand and receptor were prepared using AutoDock Tools 1.5.6 (ADT). In brief, only the dimer interface of hTLR8-hTLR8* (leucine-rich repeat 11- leucine-rich repeat 14 and leucine-rich repeat 16*- leucine-rich repeat 18*) that is known to be involved in the interaction with the ligands was selected (center x = 18, y = −22 and z = 22 and size x = 20, y = 20 and z = 20), to facilitate docking calculations. Additionally, solvent and ligand molecules were deleted and polar hydrogen and charges were added to the structure.

Docking calculations were performed for the interaction between hTLR8 and violacein or 2-(ethoxymethyl)-1H-imidazo[4,5-c]quinolin- 4-amine (CL097). All protein and ligand structures were built and saved as pdbqt format, and docking was performed with the selected region of the protein, as mentioned above. Visualization and generation of images of possible binding models for violacein or CL097 with hTLR8 were performed using UCSF Chimera^54^.

### Statistical analysis

Statistical significance of the effect of incubation with violacein (qRT-PCR and ELISA) and NF-κB fold induction was determined by analysis of variance (ANOVA). When a significant effect was found, Dunnett’s post-hoc test was used to contrast the effect of treated cells with the negative control. In cases where the data did not satisfy the normality and homoscedasticity assumptions, the data were log transformed and statistical significance was determined by ANOVA and Dunnett’s post-hoc test. A global *p* value lower than 0.05 was considered statistically significant. Results of microarray confirmation were analyzed as fold change compared to untreated cells, and a t-test was performed for each gene. A two-tailed p-value lower than 0.05 was considered statistically significant.
